# Orthorexia Nervosa Practices in Rheumatoid Arthritis: The DORA Study

**DOI:** 10.3390/nu15030713

**Published:** 2023-01-31

**Authors:** Maria Sifakaki, Konstantinos Gkiouras, Helen M. Lindqvist, Georgios Marakis, Anastasia Petropoulou, Lorenzo M. Donini, Dimitrios P. Bogdanos, Maria G. Grammatikopoulou

**Affiliations:** 1 Department of Nutritional Sciences & Dietetics, Faculty of Health Sciences, International Hellenic University, Alexander Campus, P.O. Box 141, GR-57400 Thessaloniki, Greece; 2 Unit of Immunonutrition and Clinical Nutrition, Department of Rheumatology and Clinical Immunology, Faculty of Medicine, School of Health Sciences, University of Thessaly, Biopolis, GR-41110 Larissa, Greece; 3 Department of Internal Medicine and Clinical nutrition, Institute of Medicine, Sahlgrenska Academy, University of Gothenburg, 40530 Gothenburg, Sweden; 4 Nutrition and Food Standards Unit, Hellenic Food Authority, 124 Kifisias Avenue & Iatridou 2, GR-11526 Athens, Greece; 5 Department of Experimental Medicine, Sapienza University, 00185 Rome, Italy

**Keywords:** diet, disordered eating, eating disorders, mental health, pain, obesity, remission, rheumatic disease, weight loss, DAS28

## Abstract

Medical nutrition therapy (MNT) is an indisputable component of the multidisciplinary therapeutic approach in rheumatoid arthritis (RA). Previous research has suggested that in chronic disease where nutrition is an important effector of prognosis, healthy dietary choices might take an unhealthy turn, with patients developing disordered eating in the form of orthorexia nervosa (ON). ON is characterized by a pathological preoccupation with “healthy”, “pure” eating, associated with restrictive dietary patterns, nutrient deficiencies and worsening disease outcomes. The aim of the present cross-sectional study was to evaluate ON tendencies in a sample of adult patients with RA. A total of 133 patients with RA were recruited, and completed the ORTO-15 questionnaire for the assessment of ON tendencies. Most of the patients were overweight/obese (53.4%). The results revealed ON tendencies in the sample, with the median ORTO-15 score reaching 36 (IQR: 33–39). Greater ON tendencies were associated with the female gender, and lowered ON tendencies with increasing age and body mass index. The present findings highlight the need for health professional awareness regarding the problem of ON in patients with RA and the importance of screening patients.

## 1. Introduction

Orthorexia nervosa (ON) is a feeding and eating disorder (FED) characterized by an unhealthy obsession with healthy, “clean” eating [1,2,3]. ON practices can often be very restrictive, excluding certain food groups from the diet which are deemed as “unhealthy” by the patients, leading to inadequate nutrient intake, the development of nutritional deficiencies, medical complications, distress and a variety of psychological issues [1,4,5]. A greater degree of nutritional knowledge and health-related information, the frequent use of dietary supplements, dissatisfaction with body weight and body image perception, veganism, consumption of organic foods, health preoccupation, as well as extended social media use, have all been associated with increased ON symptomatology [6,7,8,9,10,11,12,13].

Although one might argue that preoccupation with “healthy” eating is not necessarily a negative aspect of a person’s behavior, in ON this preoccupation is rather a pathological obsession, and is thus, considered unhealthy. In ON, patients are fixated on food quality, carefully balancing the nutritional value of food and its perceived purity to attain health [4,8]. Individuals with ON often term the foods they select as “correct”, “pure”, “safe”, “nutritious”, “organic”, or “acceptable”, and set strict and inflexible self-imposed rules to control their diet [1]. However, this does not necessarily ensure that the diets they choose to follow are objectively healthy. ON consists of a distinct mental health disorder, falling within the fifth edition of the Diagnostic and Statistical Manual of Mental Disorders (DSM-5) [14] category of “Feeding and Eating Disorders”, associated with reduced well-being [1] and inflexible eating patterns. The recent diagnostic criteria suggested for ON are accompanied by a pledge from experts for the inclusion of the disease in the DSM as a distinct entity [1].

In chronic diseases where medical nutrition therapy (MNT) is pertinent to a holistic therapeutic approach, healthy choices often take an unhealthy turn. Several research items have pointed to the fact that many patients with a chronic disease diagnosis or specific health conditions exhibit a pathological fixation on eating “correctly” for achieving optimal disease management [1,2,3,15]. As a result, ON has been identified among patients with type 1 and type 2 diabetes mellitus [2], celiac disease [3], as well as cancer [13,16]. In fact, according to recent case-control studies [13,16], women with a cancer diagnosis exhibited greater ON symptomatology compared to a group of apparently healthy women, and this was associated with fear of cancer recurrence due to the associated eating habits and the diagnosis of additional chronic diseases, aside from cancer.

Another chronic disease where ON tendencies should be assessed includes rheumatoid arthritis (RA). RA is a chronic autoimmune rheumatic and musculoskeletal disorder (RMD) of the joints of unknown etiology, with inflammatory arthritis being the main characteristic, in addition to a variety of extra-articular manifestations (EAMs). The gradual progression of joint inflammation activates several signaling molecules inside the osteoclasts, propelling bone resorption and cartilage erosion, resulting in the destruction of the affected joints [17,18]. Morning stiffness, musculoskeletal pain and swelling consist of common RA manifestations [17]. The modern standard of care consists of early treatment with conventional synthetic, targeted synthetic and biologic disease-modifying anti-rheumatic drugs (DMARDs), in combination with glucocorticoids (GCs) and/or non-steroidal anti-inflammatory drugs (NSAIDs) [19,20]. Although pharmacological treatment of RA has improved substantially during the past decades, there is no cure, and many patients do not reach sustained remission [21]. Non-pharmacological treatment is also of pivotal importance in the multidisciplinary approach to tamp down disease activity, comprising physical therapy, lifestyle modifications and MNT [22,23,24].

In MNT in particular, the aim for patients with RA is to confer support for the multiple disease and medication-associated nutritional complications, correct nutritional deficiencies, reduce the need for NSAIDs use and drug toxicity, and by inference, improve the quality of life (QoL) of patients [23,25]. A plethora of oral nutrient supplements (ONS) and dietary patterns have been shown to be promising and efficacious in improving disease outcomes in RA [25,26,27,28,29]; as a result, many patients are requesting a more holistic and natural approach [18]. Research has shown that in their majority, patients with an RA diagnosis adhere to diets of poor-to-moderate quality [30,31,32], either by following unbalanced diets [31], or by demonstrating low adherence to objectively healthy dietary patterns, such as the Mediterranean diet [30]. Nonetheless, patients with RA do acknowledge the importance of diet in disease management and health attainment [33]. According to research, bidirectional relationships exist between eating disorders and autoimmune diseases, based on common immunopathological mechanisms [34,35,36]. However, ON tendencies have never been assessed in patients with RA, although it is possible that they might prevail within the RA patient community.

The present cross-sectional study (Diet and Orthorexia in Rheumatoid Arthritis, DORA) aimed in evaluating ON tendencies in patients with RA.

## 2. Materials and Methods

### 2.1. Participant Recruitement

Adult patients with RA were recruited online from patient groups and forums for RA with the valuable help of the Greek RMD patient society (ELEANA https://www.arthritis.org.gr/), during the May–August of 2022, through an open call for participants. Inclusion criteria involved adult patients with RA, willing to participate in the study, and communicating in the Greek language.

### 2.2. Ethics

The study was conducted in accordance with the Declaration of Helsinki, and the study protocol was approved by the Scientific Committee of the University General Hospital of Larissa (Committee Assembly 13/27-09-2022). All patients were informed of the nature and purpose of the study and provided consent by ticking in a specific box situated at the beginning of the online form, before being transferred to the online questionnaire.

### 2.3. Tools

A structured online questionnaire was completed by all participants with information regarding demographics (educational level, gender, age, family status, employment status, manual/office labor, private/state employment, rural/urban residence, smoking status, living with others/alone and perceived income sufficiency) and disease characteristics (duration, disease status, etc.). The body weight and height of participants was self-reported, and body mass index (BMI) was calculated for all. Body weight status was assessed based on the World Health Organization BMI cut-offs for the classification of underweight (BMI < 18.5 kg/m^2^), normoweight (18.5 kg/m^2^ ≤ BMI < 25 kg/m^2^), overweight (25 kg/m^2^ ≤ BMI < 30 kg/m^2^) and obesity (BMI ≥ 30 kg/m^2^) for adults [37].

ON tendencies were evaluated using the translated and culturally adapted Greek version of the ORTO-15 questionnaire [28,29]. The ORTO-15 consists of 15 questions assessing the frequency of ON tendencies occurrence. The ORTO-15 consists of a self-reported measure designed to assess ON, with its main goal being to screen rather than diagnose ON tendencies [38]. Each of the 15 questions has four possible answers that express the frequency of occurrence in a Likert scale (always, often, sometimes and never), receiving a score ranging between 1 and 4 [39]. The total score is calculated by summing the scores of the 15 individual questions. Currently, no specific cut-off exists for diagnosing ON, using either the ORTO-15, or other screening tools. Details of the Greek version of the questionnaire have been previously reported in detail, and the tool is available for use by other researchers [40]. In the initial publication of the ORTO-15 [39], a threshold value was proposed for the diagnosis of ON. However, due to the lack of established criteria for ON, the use of thresholds to diagnose ON has been deemed “unsafe”, and researchers suggested that their use should be avoided [38].

### 2.4. Statistical Analyses

Continuous variables were described with either the mean ± standard deviation (SD), or the median with the respective interquartile range (IQR) in parentheses, when they were deemed to be normally, or non-normally distributed, respectively. Qualitative variables were reported as frequencies. Normality in the data distribution was assessed by visual inspection of normal quantile-quantile (Q-Q) plots and the use of the Kolmogorov–Smirnov test.

Comparisons between the answers to individual ORTO-15 questions and ORTO-15 total scores among patient subgroups were performed using the Mann–Whitney U test.

Logistic regressions were employed for the calculation of odds ratios (OR) and their corresponding 95% confidence intervals (CI), in order to evaluate the association between RA remission (dependent variable) and the other study variables.

In addition, a multivariable logistic model was built with remission as the dependent variable, and with the following independent variables: gender (male versus female), BMI (kg/m^2^), educational level (up to high school versus higher education), smoking status (no versus yes) and RA duration (years) [41,42], plus the ORTO-15. Age was left out of this model due to 15 missing/illegible values.

Furthermore, univariable linear regression models were applied to examine the association between the ORTO-15 score (as the dependent variable) and the rest of the study variables. Linear regression’s assumptions of normality and homoscedasticity were evaluated on the residuals. Due to violations of these assumptions –namely normality–, the 95% bias corrected and accelerated bootstrap confidence intervals (95% BCa CI) were calculated, based on 2000 resamples [43]. Linear regressions are summarized by beta coefficients (β coeff.), with their 95% standard CI and 95% BCa CI.

Finally, a zero-truncated negative binomial (ZTNB) regression model was employed to examine whether the ORTO-15 score affects the time (measured in days) in remission, or active RA. ZTNB regressions were summarized by their exponentiated coefficients, with their respective 95% CI [44].

Reliability of the ORTO-15 was assessed with the Cronbach’s alpha coefficient [45] for ordered scales, as well as with the omega coefficient [38]. A threshold of α = 0.70 is considered to be indicative of the internal consistency of the examined instrument [46].

All analyses were carried out on SPSS (IBM Corp. Released 2021. IBM SPSS Statistics for Windows, Version 28.0, Armonk, NY: IBM Corp) and the *R* language [47]. The level of significance was set at α = 0.05 for all analyses.

## 3. Results

Table 1 details the descriptive characteristics of the participants.

After the exclusion of participants with extreme/implausible values, a total of 133 patients with RA were included in the final sample, the vast majority being women, with a median BMI of 25.83 kg/m^2^. According to their reported body weight and height, most of the patients were overweight/obese (53.4%), 43.6% were normoweight and the remaining 3% were underweight (all women). The median duration of RA in the sample was 5 years. The majority of patients reported being in remission (54.9%) during the study, with the remaining reporting some symptoms of active disease, including morning stiffness, joint swelling and pain, etc. As this was an open-ended question with patients providing input on perceived symptoms, results regarding reported symptoms could not be analyzed statistically. Most of the participants reported having attained primary/secondary education (49.6%), 39.1% had university education and the remaining had a postgraduate degree. The majority of the sample abstained from cigarette smoking (63.2%), living with other family members (88.7%) and having an income that was deemed as sufficient (63.2%).

The median ORTO-15 score of the sample was 36 (IQR: 33–39). The Cronbach’s α coefficient of the tool was 0.73, and the omega coefficient was 0.79. Table 2 details the answers to each of the ORTO-15 questions between patients with active or inactive disease. No differences were noted in the individual questions, or the total ORTO-15 scores between the two groups.

Based on the univariable and multivariable logistic regression models (Table 3), none of the examined associations reached significance.

Furthermore, univariable linear regressions (Table 4) revealed an increase in the ORTO-15 score with ascending age (β coeff: 0.07, 95%BCaCI: 0.001 to 0.14; *p* = 0.037) and BMI (β coeff: 0.13, 95%BCaCI: 0.02 to 0.25; *p* = 0.020), whereas a decrease was noted in the ORTO-15 score with female gender (β coeff: −2.50, 95%BCaCI: −4.17 to −0.88; *p* = 0.002). Other variables which were non-significantly associated with greater ORTO-15 scores, including single-divorced status, years of cigarette smoking and duration of RA.

Finally, ZTNB regressions revealed a non-significant, negative association of the ORTO-15 score with the days in remission (exponentiated coeff: 0.93, 95%CI: 0.85 to 1.02, *p* = 0.150), and a significant negative association of the ORTO-15 score with the duration of RA exacerbation (exponentiated coeff: 0.90, 95%CI: 0.81 to 0.99, *p* = 0.045).

## 4. Discussion

The present study revealed that patients with RA exhibit high ON tendencies. Greater ON tendencies were associated with female gender, and lowered ON tendencies with ascending age and BMI.

Research is inconclusive on the relationship between ON and BMI [1], but previous studies in patients with chronic diseases show a positive association between BMI and ON tendencies [48]. It appears that the disease diagnosis, paired with the elevated BMI, both of which have important health consequences, place a double burden on patients with chronic diseases. In turn, this double burden may eventually initiate ON tendencies in an effort to “control” disease prognosis and improve health status. In parallel, GC use is common in RMD therapy, and is associated with greater body weight accumulation, being overweight and obese [49,50], which in turn, are related to poor body image perception, greater anxiety and body image disturbance [51,52]; all of these factors can gradually precipitate disordered eating behaviors.

A plethora of evidence highlights the disproportional increased prevalence of FEDs among women [53,54,55]. In parallel, ON has been suggested to prevail among women [40,48], as verified in the present sample, where the female gender was associated with increased ON tendencies. In general, women have been shown to harbor more body image concerns, and value appearance more compared to the men [56,57]. Women with RA in particular, have been reported to attain more negative body image perceptions, and be less satisfied with their bodies [58,59]. In this manner, women with RA appear prone to the development of FEDs, including ON.

With respect to age, relevant systematic reviews and the recent panel on the definition of ON failed to reach a consensus regarding the association of ON with specific age groups [1,2]. However, in the present sample, ON tendencies decreased with ascending age, indicating that people are mostly focused on diet when newly diagnosed. Similar findings were also suggested in patients with celiac disease [3], as well as among patients with diabetes mellitus [2,48,60], where negative relationships were observed between ON tendencies and the age of participants.

People with a chronic disease diagnosis are often affected psychologically from their diagnosis, the perceived support offered to them, the disease manifestations, the plethora of comorbidities, the reduced QoL and increased physical disability, and the restrictions associated with disease management [61,62]. In RA, high rates of depression and anxiety, a considerable amount of psychiatric comorbidities and an increased prevalence of panic attacks have been reported [63,64,65,66,67,68], and it appears that greater disease activity is associated with increased stress, physical disability, fatigue and healthcare costs [63]. According to Sturgeon [68], the underlying pathways connecting RA to mental health are, most likely, mutually influential, which stress initiating flairs and inflammation; these, in turn, may further exacerbate anxiety and depression. However, in RA, the triggering of disordered eating may in fact, constitute more than the residue of disease-related anxiety [2], attaining a physiological basis. Chronic pain alters the neural pathways of the reward process, while inducing alterations in the neurotransmission of dopamine, which can disturb behavior [68], including eating behavior. Thus, it is possible that adhering to a “clean” diet is considered as a reward for patients with an RA and comorbid ON diagnosis.

The fear of relapsing in chronic disease [16] and the fear of becoming ill [69] have both been associated with increased ON tendencies. In RA, the clinical course of the disease is characterized by frequent alternating periods of disease relapse and remission. Due to the heterogenous manifestations of the disease, remission and prognosis are difficult to predict [70]. Subsequently, contradictory factors have been associated with remission in the literature among patients with RA, including older age, female gender, obesity status, history of smoking, comorbidities, fewer EAMs, use of DMARDs, treatment with GC, poor functional status, increased disease activity and erythrocyte sedimentation rate (ESR) at the time of diagnosis [41,71,72,73]. All of the evidence points to the fact that relapse in RA is difficult to predict. More recently, artificial intelligence was used to predict RA remission, exhibiting superior performance in comparison to researcher-selected variables [74]. In the present study however, the duration of active disease was not associated with ON tendencies.

Limitations of the present study include the small sample size, especially with regard to men, which does not allow for generalization of the findings; however, it offers ample food for thought for the conduction of future research. It is possible that the inclusion of a control group with healthy participants may have provided more evidence with regard to the research question. Furthermore, a prospective cohort design may have been preferred, as it would provide insight into the risks associated with ON practices in RA. However, a prospective design is difficult to implement online, as it often leads to the recruitment of a very small sample. The online recruitment provided the opportunity to collect data from patients attending different rheumatology clinics in the country; however, several important variables could not be collected with accuracy, and were left out. These included biochemical (ESR, C-reactive protein levels, etc.), medication-related (type and doses) and disease activity data. It is possible that if medical doctors provided more objective input on disease activity and remission, that the results might have differed. Furthermore, several variables collected herein were self-reported by the patients, rather than being recorded, or directly assessed by health practitioners. Unfortunately, this may have introduced reporting bias into the results. An additional limitation of the study involves the use of the ORTO-15 tool, which has been previously criticized by some researchers. Nonetheless, given that the consensus definition of ON is relatively recent [1], the lack of a specific tool based on the exact criteria discussed in the consensus is apparent.

Future research should continue the evaluation of ON tendencies among patients with RA and, when applicable, a prospective design should be used, in order to understand the consequences of ON in distinct RA outcomes and disease prognoses. It would also be of interest to see whether pain and disease activity, measured with the disease activity score 28 (DAS-28) are related to ON tendencies. Furthermore, it would be interesting to understand if the problem is also extended to patients with other RMD diagnoses.

## 5. Conclusions

Undeniably, our relationship to different foods and habitual dietary intake have important effects on our psychological and physical well-being. Given that the promotion of healthy eating is of central importance in chronic diseases, including RA [23], it must be delivered by experts, with a parallel assessment and close follow up of each patient’s dietary modifications. The present results show that a great number of patients with RA demonstrate ON tendencies. Nonetheless, most health professionals including rheumatologists have little education on nutrition and on ON, in particular. Therefore, it is unlikely that patients with RA receive optimal care concerning dietary advice, which could lead to unnecessary stress and psychological illness. Professional education and awareness are important steps to take towards the early identification of patients with RA exhibiting ON practices.

## Figures and Tables

**Table 1:** General characteristics of the participants (N = 133).

Characteristics	Statistics *
Age (years)	46.52 ± 9.78 ^†^
Gender (male/female) (*n*)	7/126
BMI (kg/m^2^)	25.83 (22.34, 30.65)
Body weight status (underweight/normoweight/overweight/obese) (*n*)	4/58/34/37
Educational level attained (primary-secondary/tertiary/postgraduate) (*n*)	66/52/15
Smoking (no/yes) (*n*)	84/49
Smoking duration (years)	24.3 ± 9.3
RA duration (years)	5 (3, 11) ^‡^
Income (Insufficient/sufficient) (*n*)	49/84
Unemployed (*n*)	49
Accommodation (alone/with family members) (*n*)	15/118
RA on remission (*n*)	73
ORTO-15	36 (33, 39)

BMI—body mass index; IQR—interquartile range; ORTO-15—orthorexia nervosa 15-item questionnaire [28,29]; RA—rheumatoid arthritis; SD—standard deviation; * data are presented as medians with their respective IQR in parentheses, means ± SD, or as counts (*n*); ^†^ for 15 participants, data on age were missing; ^‡^ data were missing for 1 participant.

**Table 2:** Answers to each of the individual questions of the ORTO-15 questionnaire [28,29] ^†^.

	Questions *	Patients with Inactive RA (*n* = 73)	Patients with Active RA (*n* = 60)	*p* Value ^‡^
1.	Paying attention to the calories of the food while eating:	3 (2, 4)	3 (2, 4)	0.532
2.	Feeling confused when visiting food shops:	4 (3, 4)	3 (3, 4)	0.725
3.	Worrying with the thought of food during the last 3 months:	3 (2, 3.5)	3 (2, 3)	0.285
4.	Eating choices conditioned by health status:	2 (1, 3)	2 (1, 3)	0.647
5.	Considering the taste of food as more important than the quality:	3 (2, 3)	3 (2, 3)	0.507
6.	Willingness to spend more money to have healthier food:	3 (2, 3)	2 (2, 3)	0.754
7.	Worrying about food for ≥ 3 h/day:	4 (3, 4)	3.5 (3, 4)	0.691
8.	Allow oneself to perform eating transgressions:	3 (2, 3)	3 (2, 3)	0.605
9.	Belief that mood affects eating behavior:	2 (1, 3)	2 (1, 3)	0.155
10.	Belief that the conviction to eat only healthy food increases self-esteem:	2 (2, 3)	3 (2, 3.75)	0.143
11.	Belief that eating healthy food impacts one’s lifestyle (frequency of eating out, eating with friends, etc.):	2 (2, 3)	2 (1, 3)	0.140
12.	Belief that consuming healthy food may improve appearance:	1 (1, 2)	1 (1, 2)	0.877
13.	Feeling guilt when transgressing with food:	3 (2, 3)	2 (1, 3)	0.428
14.	Belief that unhealthy foods also exist on the market:	1 (1, 1.5)	1 (1, 2)	0.249
15.	Consumes meals alone at the present time:	3 (2, 3)	3 (2, 3)	0.450
Total ORTO-15 Score:		36 (33.5, 39)	35.5 (33, 38)	0.603

IQR—interquartile range; ORTO-15—orthorexia nervosa 15-item questionnaire [28,29]; RA—rheumatoid arthritis; * based on the English version of the original questionnaire; ^†^ data are presented as medians with their respective IQR in parentheses; ^‡^ based on the Mann–Whitney U test.

**Table 3:** Results of univariable and multivariable logistic regressions examining the association of study variables with reported remission status (*n* = 133).

Variable	Univariable	Multivariable
	OR (95% CI); *p* Value	OR (95% CI); *p* Value
Age (years) ^†^	1.01 (0.97 to 1.04); 0.780	-
Female gender	0.47 (0.09 to 2.51); 0.376	-
BMI (kg/m^2^)	1.00 (0.94 to 1.06); 0.910	1.00 (0.94 to 1.07); 0.948
Single/divorced status	0.95 (0.43 to 2.08); 0.892	-
Higher education	0.71 (0.36 to 1.42); 0.334	-
Unemployment status	Ref	Ref
Public/private sector employment	1.74 (0.83 to 3.63); 0.143	1.79 (0.83 to 3.83); 0.136
Manual labor employment	3.07 (0.85 to 11.13); 0.088	3.10 (0.84 to 11.47)
Living with others	1.46 (0.49 to 4.26); 0.499	
Rural residence	1.18 (0.53 to 2.67); 0.685	
Sufficient income	0.87 (0.43 to 1.76); 0.690	0.73 (0.35 to 1.53); 0.410
Smoking status	1.01 (0.50 to 2.06); 0.970	
Smoking (years)	0.95 (0.89 to 1.01); 0.115	
RA duration (years) ^‡^	0.99 (0.96 to 1.03); 0.713	
ORTO-15 (score)	1.01 (0.92 to 1.11); 0.876	

BMI—body mass index; CI—confidence intervals; OR—odds ratio; ORTO-15—orthorexia nervosa 15-item questionnaire [28,29]; RA—rheumatoid arthritis; Ref—reference category; ^†^ for 15 participants, data on age were missing; ^‡^ data were missing for 1 participant.

**Table 4:** Univariable linear regression associations of study variables with ORTO-15 score.

Variable	Regressions with Standard CI	Regressions with BCa CI
	β Coeff (95% CI); *p* Value	β Coeff (95% Bca CI); *p* Value
Age (years)	0.07 (0.003 to 0.14); 0.040	0.07 (0.001 to 0.14); 0.037
Female gender	−2.50 (−5.34 to 0.34); 0.083	−2.50 (−4.17 to −0.88); 0.002
BMI (kg/m^2^)	0.13 (0.02 to 0.24); 0.020	0.13 (0.02 to 0.25); 0.020
Single-divorced status	0.38 (−1.09 to 1.85); 0.609	0.38 (−1.10 to 1.79); 0.632
Tertiary education attainment	−0.96 (−2.23 to 0.32); 0.139	−0.96 (−2.16 to 0.39); 0.148
Unemployment status	Ref.	Ref.
Public/private sector employment	0.15 (−1.23 to 1.53); 0.829	0.15 (−1.15 to 1.52); 0.834
Manual labor employment	−0.38 (−2.62 to 1.87); 0.740	−0.38 (−2.27 to 1.49); 0.702
Living with others	−1.40 (−3.41 to 0.61); 0.170	−1.40 (−3.22 to 0.64); 0.150
Rural residence	−0.04 (−1.48 to 1.55); 0.963	−0.04 (−1.38 to 1.52); 0.957
Income perceived as insufficient	−0.16 (−1.49 to 1.16); 0.808	−0.16 (−1.53 to 1.20); 0.821
Smoking status	−0.29 (−1.62 to 1.04); 0.667	−0.29 (−1.64 to 1.11); 0.676
Smoking (years)	0.02 (−0.11 to 0.15); 0.771	0.02 (−0.10 to 0.15); 0.744
RA duration (years)	0.04 (−0.03 to 0.11); 0.302	0.04 (−0.02 to 0.10); 0.237

BMI—body mass index; (Bca) CI—(bias corrected and accelerated) confidence intervals; ORTO-15—orthorexia nervosa 15-item questionnaire [28,29]; RA—rheumatoid arthritis; Ref—reference category.

## Data Availability

Data are available upon request to the first authors.
